# Neurosurgery 4.0: A Scoping Review of Current Trends and Challenges in Specialized Smart Hospitals

**DOI:** 10.7759/cureus.92673

**Published:** 2025-09-18

**Authors:** Ehab Shabo, Sevgi Sarikaya-Seiwert, Valeri Borger, Matthias Schneider, Hartmut Vatter, Lutz Hager

**Affiliations:** 1 Department of Neurosurgery, University Hospital Bonn, Bonn, DEU; 2 Section of Pediatric Neurosurgery, Department of Neurosurgery, University Hospital Bonn, Bonn, DEU; 3 Department of Health Care Management, Stiftung Rehabilitation Heidelberg (SRH) Distance Learning University, Riedlingen, DEU

**Keywords:** artificial intelligence, neurosurgery, scoping review, smart-hospital, trends and challenges

## Abstract

The worldwide increase in neurosurgical conditions, due to global population growth and increased life expectancy, challenges healthcare systems, necessitating more precise diagnostics and personalized treatment strategies despite workforce shortages.

While rapid technological progress, particularly in the field of artificial intelligence (AI), holds significant promise for enhancing diagnostics, therapeutic strategies, and administrative efficiency in healthcare, there is a marked absence of comprehensive, field-specific analyses addressing these developments within specialized Smart Hospital environments such as neurosurgery centers. To date, the literature lacks a scoping review that synthesizes current trends, challenges, and opportunities across multiple dimensions, including technological, administrative, financial, and clinical aspects. This study presents the first scoping literature review on current AI implementation in healthcare systems, critically evaluating both its advantages and limitations, and aims to fill that gap by providing a holistic, multidisciplinary overview of the Smart Hospital concept in the context of neurosurgical care, offering a unique contribution to the existing body of knowledge.

Additionally, based on a thorough analysis of examples of the most prominent existing Smart Hospital concepts, the study concludes with conceptual considerations and future research directions derived from the literature for the development of a novel, fully AI-driven neurosurgical Smart Hospital concept.

## Introduction and background

The prevalence of neurological and neurosurgical diseases, such as brain tumors, hemorrhages, strokes, and neurodegenerative disorders, is increasing worldwide. This is due to the growing global population and rising life expectancy. The increasing prevalence of these conditions entails significant and growing challenges to healthcare systems and underscores the necessity for continuous development in diagnostic precision and individualized treatment approaches to optimize patient outcomes and improve healthcare efficiency [1]. Simultaneously, the lack of skilled professionals exacerbates the challenge, often leading to prolonged waiting times that may delay required treatments, thus compromising patients’ outcomes [2].

The rapid advancement of technology, particularly the expeditious emergence and progress of artificial intelligence (AI), holds the potential to revolutionize diagnostic and therapeutic processes while optimizing administrative workflows through automation, ultimately improving the overall efficiency and treatment quality [3-8]. Neurosurgery, in particular, benefits from the integration of emerging technologies. AI-driven image analysis has revolutionized diagnostic methods, while intelligent decision-support systems and robotic-assisted procedures have improved surgical planning and precision [9,10]. Furthermore, machine-learning algorithms enable the development of personalized treatment strategies, optimizing patient-specific therapies and enhancing clinical outcomes [6,11-14].

Some hospitals around the globe have already started implementing AI across various domains, earning them the designation of "Smart Hospitals" [15,16]. The literature offers numerous reports of AI integration in hospitals, highlighting its positive impacts on healthcare. However, field-specific, objective, and comprehensive studies that analyze the feasibility of AI implementation from a multidimensional perspective (technological, medical, financial, and administrative aspects) are currently lacking, particularly in the field of neurosurgery.

Accordingly, the guiding research question of this scoping review is “What is the current state of AI integration in neurosurgery and related hospital structures, and what implications does this hold for the development of specialized Smart Hospitals?”

To address this, the study pursues three objectives: Firstly, to map existing evidence on AI applications in neurosurgery and healthcare management; secondly, to evaluate reported benefits and challenges across technological, medical, financial, and administrative domains; and thirdly, to explore conceptual implications for future Smart Hospital models in neurosurgery.

## Review

Methods

A scoping literature search was conducted to identify studies focusing on smart hospital implementations in neurosurgical settings, addressing technological, administrative, financial, and social considerations. The databases searched included PubMed, Google Scholar, and the Cochrane Library, covering publications from January 2003 to December 2024.

The search strategy employed a combination of keywords and Boolean operators as follows: ((smart hospital) OR (digital health) OR (Health care system)) AND ((neurosurgery) OR (Specialized Hospital)) AND ((artificial intelligence) OR (AI)) AND ((advantages) OR (benefits) OR (challenges) OR (trends)) AND ((financing) OR (finance models) OR (funding) OR (payment systems))

Filters applied included English and German languages and full text availability.

Inclusion and Exclusion Criteria

Studies were included if they focused on the implementation of smart hospital technologies within neurosurgery or other specialized medical fields, characterized by advanced, highly technical procedures or focused expertise beyond general medicine, including, but not limited to, neurosurgery, cardiology, oncology, radiology, and orthopedics. These fields were selected because they share comparable requirements for precision, complex workflows, and integration of advanced technologies such as AI, robotics, and digital infrastructure. The inclusion of these disciplines allows the review to capture transferable insights and best practices relevant to neurosurgical Smart Hospitals while maintaining a focus on highly specialized care. Articles were also included if they addressed one or more of the following domains: technological, administrative, financial, or social considerations. Articles and reviews published between January 2003 and December 2024 were considered eligible. Review articles were included to enhance the scope and context rather than to inform quantitative synthesis.

Studies were excluded if they did not specifically pertain to neurosurgery, other specialized medical disciplines, or the context of smart hospitals.

Study Selection

The initial database search yielded 1,260 articles. After applying the mentioned filter strategy, 242 articles could be identified. Following the removal of duplicates and a preliminary screening of titles and abstracts for relevance, 136 articles were selected for full-text review. After applying the predefined inclusion and exclusion criteria, 52 studies were deemed eligible and included in the final analysis. To assess the overall quality of the available evidence and to provide a basis for policy development or future systematic reviews, the study selection process followed the Preferred Reporting Items for Systematic Reviews and Meta-Analyses (PRISMA) and risk of bias guidelines.

In addition to the systematic database search, a supplementary investigation was conducted to enhance the practical relevance of the review. This involved a theoretical analysis of existing global smart hospital models through the examination of publicly available hospital quality reports. The aim was to identify and evaluate conceptual frameworks, operational structures, and implementation strategies, thereby offering real-world insights into current applications of AI in specialized hospital settings.

Further targeted searches were carried out across institutional websites of leading Smart Hospitals, as well as global health organizations such as the World Health Organization (WHO), to identify relevant reports and publications addressing the integration of AI in healthcare. This supplementary search yielded 12 additional publications that met the inclusion criteria. In total, 64 references were incorporated into the final analysis.

**Figure 1:**
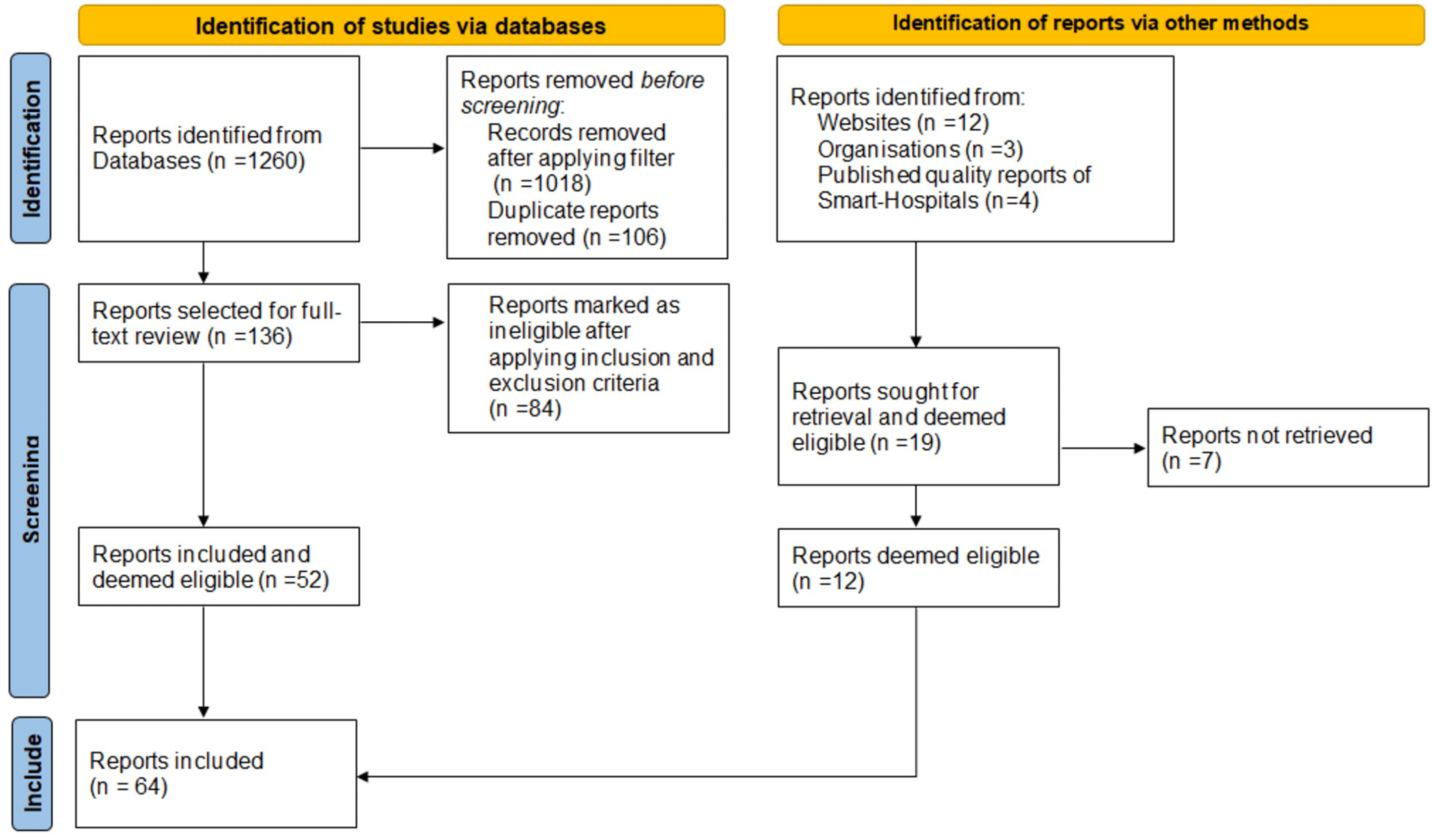
Represents the methodology and study selection of this scoping review. Preferred Reporting Items for Systematic Reviews and Meta-Analyses (PRISMA) 2020 flow diagram that describes the methodology of this systematic review (Source: [17]). To view a copy of this license, visit https://creativecommons.org/licenses/by/4.0/).

Methodology is demonstrated as a PRISMA flowchart per the latest guidelines 2020 in Figure 1.

Risk-of-Bias Assessment

We conducted a structured risk-of-bias assessment for each of the 64 included studies using the ROBINS-I framework [18].

Although formal risk-of-bias assessments are not a standard requirement for scoping reviews, we chose to apply the ROBINS-I framework to enhance transparency and provide readers with a clearer understanding of the methodological quality of the included studies. This approach allows us to systematically identify potential biases across multiple domains, supporting a more critical interpretation of the evidence and ensuring that our evaluation of AI integration in neurosurgical Smart Hospitals is grounded in a transparent appraisal of available research.

Two authors (ES and LH) independently assessed each study using the seven domains of the ROBINS-I tool: (1) bias due to confounding, (2) bias in selection of participants, (3) bias in classification of interventions, (4) bias due to deviations from intended interventions, (5) bias due to missing data, (6) bias in measurement of outcomes, and (7) bias in selection of the reported result.

Each domain was rated as Low, Moderate, Serious, Critical, or No Information (N/A). Discrepancies were resolved by discussion and, when required, adjudicated by a third reviewer (SSS). To derive an overall bias rating per study, we applied the conservative “highest‐risk” rule: the overall rating for each study equals the most severe rating assigned across its seven domains.

Drawing on the insights gained from this analysis, the study conducts a critical evaluation of the feasibility of establishing a fully AI-driven neurosurgical Smart Hospital and concludes with a set of practical recommendations to guide future developments in this emerging field.

Results

Reporting Bias Assessment

**Figure 2:**
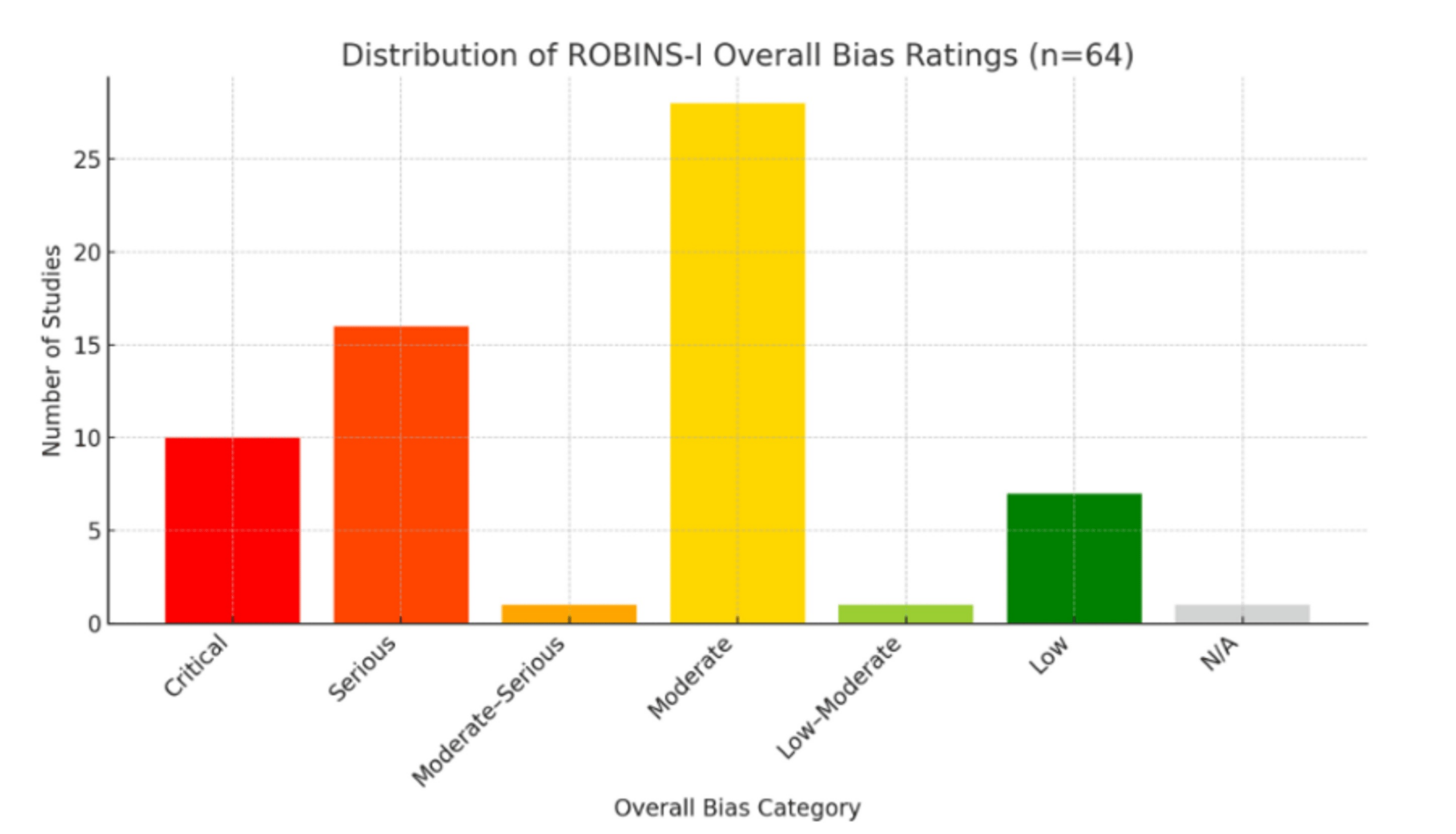
Presents the distribution of overall risk of bias assessments.

Based on the ROBINS-I tool, qualitative assessment for the 64 included studies using a traffic-light bar chart format was conducted (Figure 2). The results indicate that seven studies (10.9%) were judged to be at low risk of bias, one study (1.6%) at low to moderate risk, 28 studies (43.8%) at moderate risk, one study (1.6%) at moderate to serious risk, 16 studies (25.0%) at serious risk, and 10 studies (15.6%) at critical risk of bias. One study (1.6%) was deemed not applicable for risk of bias evaluation.

Reporting and confounding emerged as the most frequent source of bias, often due to inadequate adjustment for key variables. Full, study‐level domain ratings for all 64 references are available in Table 1.

**Table 1:** Full, study‐level domain ratings for all 64 references using ROBINS-I tool.

Serial No.	Report/Study	Study Design	Confounding	Selection	Intervention ‑ Class	Deviations	Missing Data	Outcome Measurement	Reporting Bias	Overall Bias
1	Noh SH et al. (2025) [1]	Narrative Review	Serious	Moderate	Serious	Low	Low	Moderate	Serious	Serious
2	World Health Organization (2006) [2]	Policy Report/Epidemiology	Critical	N/A	Critical	N/A	Moderate	Moderate	Critical	Critical
3	Topol EJ (2019) [3]	Perspective	Moderate	Low	Moderate	Low	Low	Low	Moderate	Moderate
4	Esteva A et al. (2021) [4]	Review With Empirical Elements	Moderate	Moderate	Moderate	Low	Low	Moderate	Moderate	Moderate
5	Monsour R et al. (2022) [5]	Descriptive Review	Moderate	Low	Moderate	Low	Low	Moderate	Serious	Moderate–Serious
6	Sudhakaran G (2024) [6]	Editorial/Mini‑Review	Serious	N/A	Serious	N/A	Low	Moderate	Serious	Serious
7	Rajkomar A et al. (2019) [7]	Perspective/Overview	Moderate	Low	Moderate	Low	Low	Low	Moderate	Moderate
8	Shameer K et al. (2018) [8]	Review	Moderate	Moderate	Moderate	Low	Low	Moderate	Moderate	Moderate
9	van Lieshout C et al. (2024) [9]	Mapping Review	Low	Low	Low	Low	Low	Low	Low	Low
10	Esteva A et al. (2017) [10]	Empirical Study	Moderate	Low	Low	Low	Low	Low	Low	Moderate
11	Senders JT et al. (2018) [11]	Systematic Review	Serious	Moderate	Moderate	Low	Moderate	Moderate	Serious	Serious
12	Lui Y et al. (2020) [12]	Narrative Review	Moderate	Low	Moderate	Low	Low	Moderate	Moderate	Moderate
13	Obermeyer Z and Emanuel EJ (2016) [13]	Perspective Study	Moderate	Low	Moderate	Low	Low	Low	Moderate	Moderate
14	Bonsanto MM and Tronnier VM (2020) [14]	Narrative Review	Moderate	N/A	Serious	N/A	Low	Moderate	Serious	Serious
15	Equans Group [15]	Institutional Website	Critical	Critical	Critical	Critical	Critical	Critical	Critical	Critical
16	Aignostics (2025) [16]	Institutional Website	Critical	Critical	Critical	Critical	Critical	Critical	Critical	Critical
17	Health IT News (2017) [19]	Journalistic Overview	Serious	Low	Serious	Low	Low	Moderate	Serious	Serious
18	Sinha R (2024) [20]	Narrative Review	Moderate	Low	Moderate	Low	Low	Moderate	Moderate	Moderate
19	Lee D and Yoon SN (2021) [21]	Narrative Review	Moderate	Low	Moderate	Low	Low	Moderate	Moderate	Moderate
20	Kazemzadeh K et al. (2023) [22]	Narrative Review	Moderate	Low	Moderate	Low	Low	Moderate	Moderate	Moderate
21	Mehrotra A & Kumar MP. (2020) [23]	Narrative Review	Moderate	Low	Moderate	Low	Low	Moderate	Moderate	Moderate
22	FMH (2022) [24]	Institutional Report	Moderate	Moderate	Moderate	Low	Moderate	Moderate	Moderate	Moderate
23	Antweiler D et al. (2024) [25]	Cross-Sectional Study	Serious	Moderate	Moderate	Low	Moderate	Moderate	Serious	Serious
24	Bures D et al. (2023) [26]	Narrative Review	Moderate	Low	Moderate	Low	Low	Moderate	Moderate	Moderate
25	OECD Report (2024) [27]	Policy Report	Critical	N/A	Critical	N/A	Moderate	Moderate	Critical	Critical
26	Bundesverband Digitale Wirtschaft Report (2024) [28]	Policy Report	Critical	N/A	Critical	N/A	Moderate	Moderate	Critical	Critical
27	Deutscher Ethikrat (2023) [29]	Policy Report	Critical	N/A	Critical	N/A	Moderate	Moderate	Critical	Critical
28	Khanna NN et al. (2022) [30]	Theoretical/Conceptual Article	Moderate	Low	Moderate	Low	Low	Moderate	Moderate	Moderate
29	Al‑Issa Y et al. (2019) [31]	Review/Survey	Moderate	Low	Moderate	Low	Low	Moderate	Moderate	Moderate
30	Torab‑Miandoab A et al. (2023) [32]	Systematic Review	Serious	Moderate	Moderate	Low	Moderate	Moderate	Serious	Serious
31	Shojaeinia M. (2024) [33]	Commentary	Serious	N/A	Serious	N/A	Low	Moderate	Serious	Serious
32	Johnson KW et al. (2018) [34]	Narrative Review	Moderate	Low	Moderate	Low	Low	Moderate	Moderate	Moderate
33	Akbar AA (2003) [35]	Qualitative Grounded Theory	Moderate	Low	Moderate	Low	Low	Moderate	Moderate	Moderate
34	Moro Visconti R and Morea D (2020) [36]	Empirical Modeling Study	Moderate	Low	Moderate	Low	Low	Moderate	Moderate	Moderate
35	Kirubakaran SJ et al. (2023) [37]	Demonstration Study	Moderate	Low	Moderate	Low	Low	Moderate	Moderate	Moderate
36	Moro Visconti R et al. (2019) [38]	Empirical Case Study	Moderate	Low	Moderate	Low	Low	Moderate	Moderate	Moderate
37	Hashemkhani Zolfani S et al. (2020) [39]	Scenario Modeling Analysis	Low–Moderate	Low	Moderate	Low	Low	Moderate	Low	Low–Moderate
38	Miller FA and French M (2016) [40]	Empirical Case Study	Moderate	Low	Moderate	Low	Low	Moderate	Moderate	Moderate
39	Liu F et al. (2023) [41]	Observational Cohort/Cross-Sectional Study	Serious	Moderate	Moderate	Moderate	Moderate	Moderate	Serious	Serious
40	Ng C‑W et al. (2021) [42]	Case Study	Serious	Moderate	Serious	Moderate	Moderate	Moderate	Serious	Serious
41	Xiang L et al. (2022) [43]	Quasi‑Experiment	Serious	Moderate	Moderate	Moderate	Moderate	Moderate	Serious	Serious
42	Wang Y et al. (2021) [44]	Observational Cohort/Cross-Sectional Study	Serious	Moderate	Moderate	Moderate	Moderate	Moderate	Serious	Serious
43	Vlaanderen FP et al. (2019) [45]	Systematic Review	Serious	Moderate	Moderate	Low	Moderate	Moderate	Serious	Serious
44	Tencent’s AI (2019) [46]	Journalistic Report	Critical	Critical	Critical	Critical	Critical	Critical	Critical	Critical
45	Ernst & Young Nederland LLP (2023) [47]	Consolidated Annual Report	Critical	N/A	Critical	N/A	Moderate	Moderate	Critical	Critical
46	Karolinska Annual Report (2024) [48]	Hospital Annual Report	Critical	N/A	Critical	N/A	Moderate	Moderate	Critical	Critical
47	Forging New Paths Report (2024) [49]	Interview/Report	Serious	N/A	Serious	N/A	Moderate	Moderate	Serious	Serious
48	Wilmer, Johns Hopkins Medicine, Annual Report (2023) [50]	Hospital Annual Report	Critical	N/A	Critical	N/A	Moderate	Moderate	Critical	Critical
49	Litjens G et al. (2017) [51]	Systematic Survey Review	Serious	Moderate	Moderate	Low	Moderate	Moderate	Serious	Serious
50	Edelson DP et al. (2024) [52]	Observational Comparative Study	Moderate	Low	Low	Low	Low	Low	Moderate	Moderate
51	Zeineldin RA et al. (2024) [53]	Empirical Systematic Study	Moderate	Low	Moderate	Low	Low	Moderate	Low	Moderate
52	Fischer N et al. (2023) [54]	Development Study	Moderate	N/A	Moderate	N/A	Low	Moderate	Low	Moderate
53	El‑Hajj VG et al. (2023) [55]	Bibliometric Analysis	N/A	Low	N/A	N/A	N/A	N/A	Moderate	Moderate
54	Jordan MI and Mitchell TM (2015) [56]	Perspective Review	Low	Low	N/A	N/A	Low	Low	Low	Low
55	Zeineldin RA et al. (2023) [57]	Feasibility Study	Moderate	Low	Moderate	Low	Moderate	Moderate	Low	Moderate
56	Wagner M et al. (2022) [58]	Narrative Chapter	N/A	N/A	N/A	N/A	N/A	N/A	N/A	N/A
57	Kose U et al. (2024) [59]	Monography Review	Low	Low	N/A	N/A	Low	Low	Low	Low
58	Kaissis G et al. (2021) [60]	Empirical Machine-Learning Study	Moderate	Moderate	Low	Low	Low	Low	Low	Moderate
59	Yu KH et al. (2018) [61]	Review	Low	Low	N/A	N/A	Low	Low	Low	Low
60	Warnat‑Herresthal S et al. (2021) [62]	Experimental Machine-Learning Study	Moderate	Moderate	Low	Low	Low	Low	Low	Moderate
61	Kus K et al. (2022) [63]	Cross-Sectional Study	Serious	Serious	N/A	N/A	Moderate	Moderate	Serious	Serious
62	He J et al. (2019) [64]	Perspective Review	Low	Low	N/A	N/A	Low	Low	Low	Low
63	Jiang F et al. (2017) [65]	Review	Low	Low	N/A	N/A	Low	Low	Low	Low
64	Palaniappan K et al. (2024) [66]	Review Policy Analyses	Low	Low	N/A	N/A	Low	Low	Low	Low

Characteristics and Challenges of Specialized Smart Hospitals

A Smart Hospital is a highly digitized medical facility that utilizes cutting-edge information and communication AI technologies to optimize and enhance infrastructure, clinical workflows, and management systems. Its primary goal is to provide advanced services and insights that were previously impossible, aiming for more efficient, personalized, and safer patient care while optimizing overall operational effectiveness [15,19-23].

The characteristics of current Smart Hospitals are described in Table 2. These characteristics can be fully or partially distributed across various departments within a Smart Hospital.

**Table 2:** Summary of key characteristics of current Smart Hospitals worldwide. The information presented in this table is derived from multiple sources [15,19-26]. AI: Artificial Intelligence; CT: Computed Tomography; MRI: Magnetic Resonance Imaging Table Credits: Ehab Shabo

Characteristics	Description
1. Digital Connectivity and Interoperability	All hospital systems (information systems, medical devices, patient records) are interconnected for seamless data exchange.
2. Automation	AI-driven diagnostic tools (e.g., MRI/CT image analysis) and automated processes (patient triage, surgical planning, medication management).
3. Robotics	The use of advanced surgical robots (e.g., Da Vinci, Exoscope) to enhance precision in surgical procedures.
4. Telemedicine	Remote consultations and continuous patient monitoring through telemedicine platforms.
5. Big Data Analytics	Big Data is utilized to predict disease progression and personalize treatment decisions.
6. Predictive Medicine	AI-based early warning systems and real-time monitoring to prevent complications and optimize medical care.
7. Internet of Things (IoT)	Connected sensors, wearables, and smart beds enable continuous patient monitoring and real-time data collection.
8. Smart Infrastructure	Smart building technologies for efficient energy and resource management (e.g., automated climate and lighting control).
9. Cybersecurity and Data Protection	Strict security measures, including blockchain technology for secure medical data storage and integrity.
10. Sustainability and Resource Efficiency	Energy-efficient technologies reduce ecological footprints, while digital processes reduce paper use and transport.

Numerous studies reported substantial positive impacts of AI in the healthcare system on improving efficiency, reducing costs, and enhancing treatment quality [24-26]. However, AI implementation involves several challenges [20,27-33], which can be divided into three main categories as presented in Table 3.

**Table 3:** Current challenges of AI implementation in healthcare system. The information presented in this table is derived from multiple sources [20,27-33]. AI: Artificial Intelligence; IT: Information Technology Table Credits: Ehab Shabo

Category	Challenges	Description
Technological Challenges	Interoperability and System Integration	Diverse hospital systems and lack of standardization hinder integration, causing delays and errors.
	Data Security and Privacy	Large amounts of medical data raise security concerns; cyber threats require compliance and regular audits.
	Technological Obsolescence	Rapid advancements make systems outdated quickly, requiring frequent upgrades, especially in high-investment special fields like neurosurgery.
	Technology Adoption and Staff Training	New technologies require training, particularly challenging for older staff or specialists unfamiliar with innovations.
Economic Challenges	High Investment Costs	Robotics and IT infrastructure are expensive, especially in specialized fields like neurosurgery.
	Ongoing Operational Costs and Maintenance	Technologies need updates, security measures, and maintenance, creating financial burdens.
	Cost-Benefit Ratio	Financial benefits of Smart Hospitals are difficult to quantify, despite efficiency improvements.
	Financial Uncertainties	Political and regulatory changes make long-term financial planning unpredictable.
	Lack of Funding and Public Support	Limited government funding forces hospitals to rely on private investors or loans, increasing financial dependency.
	Lack of Cost Transparency	Without clear cost breakdowns, hospitals struggle with resource allocation and savings.
	Difficult Scalability	Large-hospital technologies may require costly adjustments to fit smaller institutions.
Regulatory and Ethical Challenges	Unclear Legal Frameworks	Lack of unified AI regulations creates uncertainties in approval and application.
	Certification	AI systems need specific certification and current approval processes are not designed for them.
	Liability	Unclear responsibility for AI-related medical errors - developer, doctor, or hospital management?
	Bias and Discrimination	AI may disadvantage certain patient groups if trained on non-representative data.
	Acceptance of AI	Both staff and patients must accept and trust AI applications.

Financial Models and Payment Systems in Smart Hospitals

Financing a Smart Hospital is a key challenge, addressed through various business models involving both public and private funding sources. Modern models, such as pay-per-use or subscription options for AI solutions, offer additional alternatives [34-40]. Each model has its advantages and disadvantages. Current models used to finance a specialized Smart Hospital are presented in Table 4.

**Table 4:** Summary of the different financing models used in specialized Smart Hospitals worldwide with their key features and advantages. The information presented in this table is derived from multiple sources [34-40]. AI: Artificial Intelligence; IT: Information Technology; PPPs: Public-Private Partnerships Table Credits: Ehab Shabo

Financing Model	Key Features	Advantages	Challenges
Public Financing	Government grants, long-term strategic planning, social objectives	Ensures equitable access, supports rural areas, regulated quality standards	Bureaucratic, slow implementation, limited funding
Private Financing	Venture capital, corporate partnerships, PPPs, innovation incentives	Flexible, fast innovation, market-driven efficiency	Profit-focused, may neglect public welfare, potential higher costs
Partnerships With Technology Companies	Collaboration with tech companies (e.g., Google Health, IBM Watson)	Shared risk, access to cutting-edge technology with continuous development	Dependence on corporate interests, data privacy concerns
Pay-Per-Use	Payment per actual use of AI services	Cost-efficient, low entry barrier, scalable	Costs increase with usage, unpredictable expenses
Subscription	Fixed recurring payments for AI solutions	Predictable budgeting, continuous updates & support	Long-term financial commitment, potential underutilization
Hybrid Models	Combination of public and private funding models	Risk-sharing, scalable, innovation-friendly	Complex coordination, challenging balance between public and private interests

In specialized medical fields such as neurosurgery, payment systems play a crucial role in regulating reimbursement for highly specialized services. Due to the complexity of diagnoses and treatment methods in such disciplines, standard case-based systems like Diagnosis-Related Groups (DRG) may not adequately reflect the true costs of care. Therefore, it is essential to adapt the reimbursement system to account for the unique requirements of neurosurgical procedures, advanced medical technologies, and intensive postoperative care. Thus, alternative or modified reimbursement systems have emerged to ensure appropriate compensation for specialized treatments [36-45]. The modern worldwide payment systems used in Smart Hospitals are presented in Table 5.

**Table 5:** Summary of the payment systems used in current Smart Hospitals with its key features, advantages and limitations. The information presented in this table is derived from multiple sources [36-45]. Table Credits: Ehab Shabo

Payment System	Key Points	Advantages	Challenges
DRG (Diagnosis-Related Groups)	Case-based, flat-rate payments based on diagnosis, severity, and treatment efforts	Provides predictable payments and encourages efficiency.	May undervalue complex or prolonged cases.
PAE (Per-Diem System)	Fixed rate per treatment day, commonly used in acute care settings.	Simple to administer, transparent for routine cases.	Does not reflect the actual treatment complexity or specific costs.
Case-Mix System	Payment calculated based on the complexity and mix of cases treated, ideal for specialized hospitals by considering differences in treating complex conditions.	Tailored for specialized care; accounts for case complexity.	Consistent application may be challenging due to varying case data and analysis requirements.
DRG - P4P (Pay for Performance)	An evolved DRG model that also rewards quality and efficiency by incorporating performance metrics (e.g., patient satisfaction, guideline adherence).	Incentivizes high-quality and efficient care.	Measuring quality consistently can be complex and may be prone to metric manipulation.
Global Budgets/Global Payment System	Lump-sum payment provided for all patients over a specific period or within a defined patient group	Encourages preventive care and long-term health outcomes; simplifies budgeting.	Risk of budget overruns if patient numbers or treatment complexity exceed initial estimates.
Fee-for-Service (FFS)	Payment is made for each individual service or treatment provided, ensuring each procedure is accounted for.	Reflects the actual cost of services provided; rewards each service rendered.	May incentivize over-treatment and increased service volume over quality care.
Health Outcome-Based Payment (HOBP) and Pay for Quality (P4Q)	Links payments directly to patient outcomes and treatment success, rather than solely to the number or cost of services rendered.	Rewards successful treatment outcomes and effective patient recovery.	Requires accurate, long-term outcome measurements which can be complex and resource-intensive.

Following the consolidation of existing knowledge on the technological and financial characteristics of Smart Hospitals, it is both logical and necessary to identify and analyze existing Smart Hospital implementations worldwide with respect to these key features.

An overview of examples among the most prominent Smart Hospitals worldwide, including details about their AI application areas, investment models, and reimbursement systems, compiled from annual hospital reports, is presented in Table 6.

**Table 6:** Ten examples of most prestigious Smart Hospitals worldwide. The information presented in this table is derived from multiple sources [16,46-50]. AI: Artificial Intelligence; DRG: Diagnosis-Related Groups; EU: European Union; FFS: Fee-for-Service; NIH: National Institutes of Health; PPP: Public-Private Partnerships; P4P: Pay-for-Performance; P4Q: Pay-for-Quality; VR: Virtual Reality Table Credits: Ehab Shabo

Smart Hospital (Country)	Field of AI Implementation	Financing Model	Payment System
Mayo Clinic (USA)	AI-based diagnostics in pathology; robotic-assisted neurosurgery	Equity, foundation funding, PPP with Google Health	FFS, DGR-P4P
Cleveland Clinic (USA)	Predictive AI models for stroke; robotics for brain surgeries and navigation	Research funding, collaborations with tech companies, self-financing	DRG-P4P
National Neuroscience Institute (Singapore)	AI-based image analysis, robotics, telemedicine	Government funding, PPP with IBM Watson	Hybrid model (government funding + DRG)
Charité - Universitätsmedizin Berlin (Germany)	Pathology, AI-based MRI analysis, machine learning in neurosurgery	Government research grants, EU funding, industry partnerships	DRG, performance-based bonuses
Karolinska Institutet (Sweden)	AI for surgical planning, VR-based neurosurgeon training	EU research funding, crowdfunding, collaboration with MedTech companies	Global budget system, performance-based compensation
Peking Union Medical College Hospital (China)	AI diagnostics, big data for therapy planning	Government investments ("Healthy China 2030")	Case-Mix system, P4Q
Toronto Western Hospital (Canada)	AI for neurosurgery planning, deep learning for imaging	Government funding, private investors, AI funding programs	Mix of DRG and government funding
Tokyo University Hospital (Japan)	AI-based image analysis, automated patient monitoring	Government innovation funding, collaborations with Sony Health	DRG
Tencent AI Hospital (China)	AI-based neurological diagnostics, digital patient records	Government investments ("Healthy China 2030")	Case-Mix system, P4Q
Johns Hopkins Hospital (USA)	AI-based radiology, predictive analysis for brain tumors	NIH research grants, self-financing, collaborations with tech companies	FFS, P4P, Value-Based Care

Discussion

This study explores the evolving landscape of Smart Hospitals worldwide, focusing on the integration of AI in healthcare, particularly in neurosurgery.

Risk of Bias Assessment in Context

The distribution of risk of bias ratings across the 64 included studies aligns with findings from other systematic reviews utilizing the ROBINS-I tool in the context of non-randomized and emerging medical technologies. In our review, only 10.9% of studies were rated at low risk, while the majority were classified as having moderate (43.8%) or serious (25.0%) risk of bias. This pattern is consistent with prior literature in fields such as digital health, AI in clinical decision-making, and surgical innovations, where methodological challenges, particularly related to confounding, outcome measurement, and reporting transparency, frequently elevate the risk of bias.

AI in Neurosurgery: Trends and Limitations

Advancements in automation and robotics are revolutionizing neurosurgical procedures. AI-powered robotic assistance systems enhance precision and stability during procedures by providing real-time feedback, minimizing surgery duration, reducing complications, and improving long-term outcomes [6,14]. In addition, AI enables more accurate diagnoses and personalized treatment plans, with neural networks helping to precisely delineate tumor boundaries and robotic systems ensuring greater accuracy in minimally invasive surgeries [51-54]. These integrated AI solutions not only support complex decision-making but also enhance efficiency, minimize errors, and contribute to a safer, more patient-centered care. [20-22].

El-Hajj et al. examined in their bibliometric analysis the most highly cited and impactful publications on AI in neurosurgery and found that spine, endovascular, and neuro-oncology were the most represented fields regarding published reports on AI in neurosurgery, with AI primarily used for prediction modelling, diagnostics, and imaging [55]. However, integration of AI in other fields of neurosurgery (trauma, functional neurosurgery, pediatric neurosurgery, and endoscopic neurosurgery) was also reported [55], which reflects the increasing interest in AI implementation in neurosurgery.

Despite its significant potential, the integration of AI in neurosurgery is subject to notable limitations [56]. Furthermore, AI systems are inherently dependent on historical data, which may reduce their effectiveness in handling rare or atypical cases [10].

This represents a significant challenge, particularly in neurosurgery, where anatomical variations and complex pathologies are prevalent. Moreover, the accuracy of AI algorithms is directly influenced by the quality and comprehensiveness of their training data, restricting their applicability in less-documented fields.

While AI can serve as a valuable tool in clinical decision-making, it cannot replace the technical expertise, clinical experience, and intuitive judgment of neurosurgeons [57]. The ability to navigate complex ethical considerations in patient care remains a uniquely human responsibility [58]. Consequently, the successful implementation of AI requires not only technological refinement but also the trust and acceptance of medical professionals.

Additionally, it is crucial to acknowledge that AI-driven decision-making in neurosurgery is fundamentally shaped by human-collected data and published sources, which often contain conflicting perspectives. As a result, AI’s "intelligence" is still intrinsically linked to human interpretation, reinforcing the need for continuous validation and oversight in its clinical application.

In addition to the technical limitations, the potential for errors in AI-driven neurosurgical applications carries significant ethical and legal implications. Inaccuracies in AI predictions or recommendations, whether due to incomplete data, algorithmic bias, or unexpected patient-specific factors, could result in adverse outcomes. This raises questions regarding liability, including whether responsibility lies with the AI developers, the clinical team, or the hospital administration. Transparent documentation of AI decision-making, continuous validation against clinical standards, and clear protocols for human oversight are essential to mitigate risks. Moreover, integrating ethical frameworks and legal guidelines into AI implementation strategies is crucial to ensure patient safety, maintain trust, and support responsible clinical use.

Administrative Aspects of AI in the Healthcare System

Beyond the operating room, AI-driven automation streamlines administrative processes such as data collection, scheduling, and patient intervention tracking [26]. These advancements contribute to the development of highly automated hospital systems that alleviate staff workload while simultaneously enhancing patient safety and the overall quality of care [59,60]. However, alongside these benefits, AI adoption presents substantial technical, economic, and ethical challenges that must be addressed to ensure its responsible and effective implementation.

A key requirement for a successful AI integration is the modernization of hospital IT infrastructure, investment in workforce training, and the reorganization of workflows to maintain high standards of patient care [21,23,61]. Furthermore, regulatory and ethical considerations, particularly concerning data security and accountability, remain central to the debate on AI integration in healthcare systems [59]. Methods such as federated learning help reduce data privacy risks by allowing AI models to be trained locally without transferring sensitive patient data beyond institutional boundaries [60].

The successful implementation of AI also requires overcoming resistance from medical professionals and patients through comprehensive staff training with regular workshops and clear communication on AI’s benefits and limitations [62]. Additionally, dedicated change management teams can facilitate smooth transitions. Moreover, building patient trust through transparency in data privacy is equally vital to ensure the success of AI integration [63].

This underscores that only through transparent identification and addressing of current AI challenges can a responsible and successful AI integration be facilitated, leading to enhanced efficiency, security, and quality of patient care.

Financial Aspects of AI in the Healthcare System

The integration of AI in hospitals marks a significant technological and financial milestone that necessitates a comprehensive economic and feasibility analysis [40]. A critical component of this analysis is the cost-benefit evaluation, which compares the high initial and ongoing investments with the long-term savings and efficiency gains from AI-driven automation and digitalization [39]. Furtherly, the financial sustainability of Smart Hospitals is closely linked to their reimbursement structures [30,64-66].

The ideal financing model for developing a neurosurgical Smart Hospital must ensure financial sustainability, resource efficiency, and high-quality patient outcomes [39,40]. Due to the complexity and high costs of neurosurgical procedures, a hybrid approach that integrates multiple funding models tends to be the most effective model. This could include a combination of public-private partnerships, partnerships with IT companies, and venture capital, which can provide the necessary resources for infrastructure development and technological advancements, ensuring long-term viability and innovation. Simultaneously, a hybrid financing model implies a distribution of risk among stakeholders, which could potentially reduce the financial burden on individual institutions, support long-term sustainability, encourage innovation, and enhance the overall feasibility of Smart Hospital implementation.

Traditional payment systems like DRG and per-diem payments provide predictability and simplicity but do not fully account for the complexities of neurosurgical procedures [36-41]. More specialized models, such as the Case-Mix system, seem to offer a more precise allocation of resources by considering variations in case complexity [42]. Other approaches come with their own advantages and limitations presented in Table 4.

In conclusion, while outcome-based models prioritize clinical results over service volume, promoting efficiency and improved patient care, their implementation and cost management can be particularly challenging in a highly complex field like neurosurgery. This is due to the nature of many neurosurgical conditions, such as high-grade brain tumors, intracerebral bleeding, and malignant cerebral infarction, which often have inherently unfavorable prognoses and limited clinical outcomes despite the available advanced treatments. On the other hand, disregarding outcome-based payment systems could incentivize excessive treatments driven by financial gain rather than patient well-being. Therefore, a hybrid approach that combines cost control with quality incentives, such as integrating Case-Mix with a model from outcome-based payments, can provide the most balanced solution for neurosurgical Smart Hospitals, ensuring both financial stability and high-quality patient outcomes.

Current State of Smart Hospitals

Several hospitals worldwide have begun integrating AI into various areas, earning the title of “Smart Hospitals” with several characteristics detailed in Table 1.

These characteristics can be fully or partially distributed across various departments within a Smart Hospital. However, no hospital or medical institution can be considered entirely "smart" or exclusively AI-based. Rather, Smart Hospitals are evolving at different stages, with AI technologies integrated into specific diagnostic or treatment processes.

Furthermore, it is important to note that there are currently no standardized or universally accepted criteria for what constitutes a Smart Hospital. In principle, any hospital employing high-tech solutions could be labelled as “smart,” regardless of its overall digital maturity or systemic integration of such technologies. This ambiguity allows the term to be used broadly and inconsistently, often without oversight from regulatory or institutional bodies, which complicates objective evaluation, comparison, and policy development in this domain.

Additionally, there is a lack of transparency and published reports regarding the cost-benefit analysis and economic feasibility of AI integration from the currently available Smart Hospitals.

A further limitation of this study is the reliance on publicly available hospital quality reports and institutional websites to capture practical insights into AI implementation in Smart Hospitals. While these sources provide valuable real-world perspectives, they are not peer-reviewed and may contain marketing-oriented content. This approach was necessary due to the limited availability of peer-reviewed literature addressing the multidimensional implications of AI integration in specialized hospital settings, and the potential bias is acknowledged.

Conceptual Considerations and Future Directions

The rapid development of AI is reshaping healthcare systems worldwide. While technological progress offers opportunities for more precise, efficient, and safer medical procedures, the literature also highlights ethical, regulatory, and economic challenges that require careful attention. The findings of this review point to several important considerations for future work. Studies emphasize the importance of raising professional and public awareness about AI in healthcare, for instance, through targeted education and transparent communication about potential benefits and risks. Literature also suggests that institutional structures such as dedicated AI-focused units, ideally linked to quality management, could play a role in supporting technological oversight, cybersecurity, and system updates. Collaborative approaches, including inter-hospital exchanges and workshops, are frequently highlighted as valuable for sharing experiences and addressing common challenges. At the same time, research underscores that healthcare professionals’ acceptance of AI depends on transparent training opportunities and structured change-management strategies that help build trust. Ethical and regulatory frameworks must continue to adapt dynamically to address unresolved issues such as liability, accountability, and bias, while economic considerations, such as cost-benefit analyses of AI systems, may benefit from greater transparency but require further study to determine feasibility and impact. Finally, the review identifies a strong need for continued research to assess the long-term clinical implications of AI, validate its real-world effectiveness, and explore practical barriers to implementation, particularly in specialized fields such as neurosurgery.

## Conclusions

The vision of a fully AI-driven, functional Neurosurgery-Smart Hospital is not only within reach but represents a transformative shift in how neurosurgical care could be delivered in the near future. By integrating advanced AI across clinical workflows, surgical planning, intraoperative assistance, and patient monitoring, such a system has the potential to enhance precision, efficiency, and patient outcomes. However, realizing this vision demands more than just technological innovation as it calls for thoughtful, multidisciplinary collaboration, strong ethical frameworks, infrastructure development, and long-term strategic planning. Ensuring that all stakeholders, including clinicians, engineers, administrators, and policymakers, work in alignment will be essential for success. This study underscores the importance of viewing AI not as a standalone tool but as a foundational element in reshaping neurosurgical ecosystems for the better.
